# Comprehensive Management of Bowel Endometriosis: Surgical Techniques, Outcomes, and Best Practices

**DOI:** 10.3390/jcm14030977

**Published:** 2025-02-03

**Authors:** Angie Tsuei, Farr Nezhat, Nikki Amirlatifi, Zahra Najmi, Azadeh Nezhat, Camran Nezhat

**Affiliations:** 1 Camran Nezhat Institute Center for Minimally Invasive and Robotic Surgery, Woodside, CA 94061, USA; angie.tsuei@camrannezhatinstitute.com (A.T.); nikki.amirlatifi@camrannezhatinstitute.com (N.A.); zahranaj@yahoo.com (Z.N.); azzie@camrannezhatinstitute.com (A.N.); 2 Weill Cornell Medical College, Cornell University, New York, NY 10065, USA; farr@farrnezhatmd.com; 3 Gynecology/Oncology, NYU Long Island School of Medicine, Mineola, NY 11501, USA; 4 Stanford University Medical Center, Palo Alto, CA 94305, USA; 5 University of California San Francisco, San Francisco, CA 94143, USA

**Keywords:** bowel endometriosis, surgical management, shave excision, segmental resection, disc excision, appendectomy, gastrointestinal symptoms, rectosigmoid colon, laparoscopic surgery, complications, robotic surgery

## Abstract

Bowel endometriosis is a complex condition predominantly impacting women in their reproductive years, which may lead to chronic pain, gastrointestinal symptoms, and infertility. This review highlights current approaches to the diagnosis and management of bowel endometriosis, emphasizing a multidisciplinary strategy. Diagnostic methods include detailed patient history, physical examination, and imaging techniques like transvaginal ultrasound (TVUS) and magnetic resonance imaging (MRI), which aid in preoperative planning. Management options range from hormonal therapies for symptom relief to minimally invasive surgical techniques. Surgical interventions, categorized as shaving excision, disc excision, or segmental resection, depend on factors such as lesion size, location, and depth. Shaving excision is preferred for its minimal invasiveness and lower complication rates, while segmental resection is reserved for severe cases. This review also explores nerve-sparing strategies to reduce surgical morbidity, particularly for deep infiltrative cases close to the rectal bulb, anal verge, and rectosigmoid colon. A structured, evidence-based approach is recommended, prioritizing conservative surgery to avoid complications and preserve fertility as much as possible. Comprehensive management of bowel endometriosis requires expertise from both gynecologic and gastrointestinal specialists, aiming to improve patient outcomes while minimizing long-term morbidity.

## 1. Case Studies

Case 1: Appendectomy, cecectomy, and small-bowel endometriosis

**Scheme 1:**
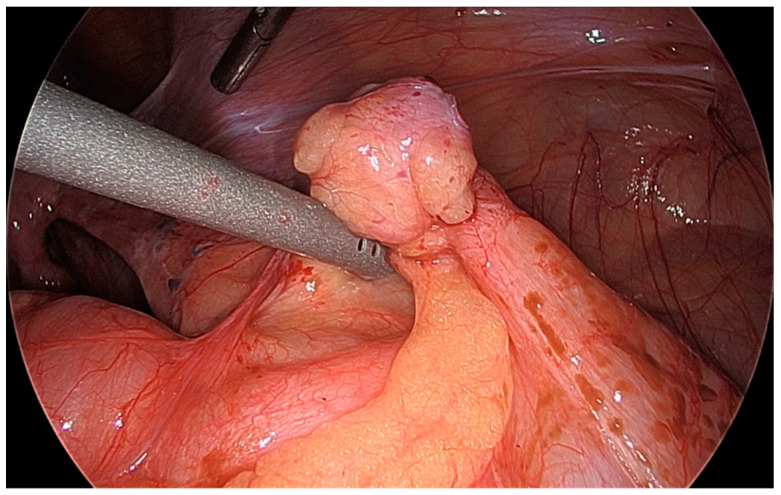
Ileal obstruction.

We present this patient as an example of an individual who would possibly benefit from a bowel resection. The patient presented as a 23-year-old with dysmenorrhea, nausea and vomiting around mittelschmerz, and dyspareunia for over a decade. She was diagnosed with endometriosis during a laparoscopic procedure two years prior. Her history was significant for prior operative laparoscopic treatment of endometriosis and bilateral ovarian cystectomies for bilateral ovarian endometriomas. She had tried and failed hormonal treatment. On bedside ultrasound, she was noted to have a small right ovarian endometrioma and uterosacral nodularity. During video laparoscopy, the patient was noted to have a frozen pelvis. She had extensive endometriosis, adhesions and fibrosis of the anterior and posterior cul-de-sac, rectovaginal septum, uterosacral cardinal ligament complex, serosa of both ureters, and also endometriosis of the serosa of the right and left external iliac artery and vein. She had endometriosis as well as dense adhesions affecting the large and small bowel to the right and left adnexa. There was endometriosis involvement of the right upper quadrant and the appendix as well as the ileocecal area as shown in Scheme 1. The endometriosis involvement caused a stricture in the ileocecal area. Due to the abnormality of the appendix, appendectomy without cecectomy was performed during the procedure. Endometriosis of the ileocecal area was shaved without entering the lumen of the bowel. This patient would be managed postoperatively with hormonal therapy. If she did not respond, she would require an ileocecectomy.

Case 2: Intussusception

**Scheme 2:**
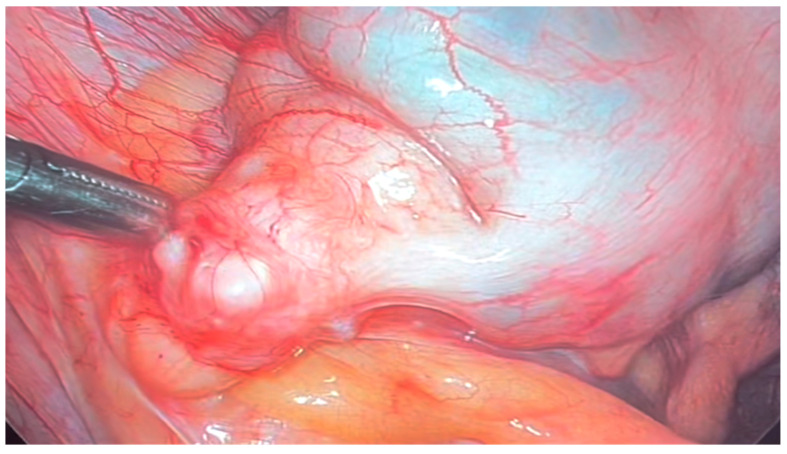
Intussusception.

We present the following patient as another example of an individual who would be an appropriate candidate for appendectomy as well as ileocecectomy. Bowel resection presents a significant risk to the patient and benefits should be weighed against the risk prior to proceeding. We present these cases to give examples of the severity of the types of cases that may warrant bowel resection. This particular patient presented as a 34-year-old with long-standing pelvic and abdominal pain since menarche. She had exhausted conservative therapies, including combined oral contraceptive pills that were somewhat controlling her pain. She was attempting to conceive and failed multiple cycles of IVF. During this time, she had stopped her combined oral contraceptive medications and her pain worsened. Her history was complicated by severe pelvic endometriosis, which required multiple previous abdominal surgeries including multiple bilateral ovarian cystectomies. Her history was also complicated by abnormal uterine bleeding, gastrointestinal symptoms, and genitourinary symptoms. At her most recent exam, she had bilateral ovarian endometriomas noted on pelvic ultrasound. The severity of her pain and failure of conservative management prompted her to seek surgical management. Intraoperatively, she was noted to have stage IV endometriosis. Her appendix was abnormal with evidence of appendiceal intussusception to the cecum and endometriosis affecting the wall of the cecum as shown in Scheme 2. Appendiceal intussusception, which is not uncommon amongst our patient population, is a condition where the appendix invaginates into the cecum. A review of the literature does not show a consistent etiology or method of treatment. However, in this patient, who presented with severe pelvic and abdominal pain and pathology confirmed endometriosis, if her pain persists, it is recommended she undergo an MRI and CT with contrast to evaluate the large and small bowel to rule out stricture. Colonoscopy is also recommended. She should be managed according to the findings.

Case 3: Ileocolic resection

Another instance of significant ileocecal disease is noted by Drs Farr Nezhat et al. in their video regarding minimally invasive ileocolic resection for deep infiltrating endometriosis (Society of Pelvic Surgeons 2024. *“Minimally Invasive Ileocolic resection for Deep Infiltrative Endometriosis: Ileocolic Resection”*) [1]. Their first case regarding a 38 yo patient with cyclic GI-dominant symptoms who exhibited endometriosis of the ileocecum. She presented with cyclic nausea, vomiting, and bloating. She also had a history of one small-bowel obstruction involving the ileum that had been managed conservatively. She had one prior robotic-assisted video laparoscopy treatment of endometriosis. MRI imaging showed a 3.5 cm endometriotic lesion involving the terminal ileum. Due to the significantly decreased quality of life and exhaustion of conservative methods, the decision was made to proceed with video-laparoscopic treatment of pelvic and bowel endometriosis. Examination of the abdomen and pelvis during surgery showed multiple endometriotic lesions in the anterior, posterior, and bilateral sidewalls. The ileocecal lesion was identified and noted to be greater than 3 cm and affecting more than one-third of the lumen. Consistent with our recommendations as further described later in this article, the affected ileocecum was resected. In this situation, a segmental bowel resection was appropriate.

Case 4: Obliterated posterior cul-de-sac

**Scheme 3:**
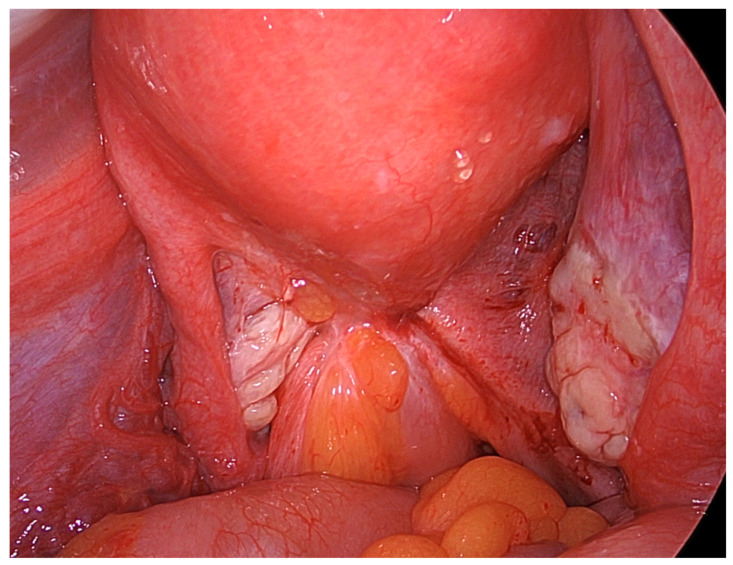
Cul-de-sac posterior obliteration.

Lastly, we present a case where shaving of the bowel was appropriate. In this case, the 31-year-old had been referred for fertility concerns in the context of multiple fibroids. It was incidentally noted during her initial evaluation that she had cyclic diarrhea, nausea, vomiting, and frequency of bowel movements. Her history was further complicated by polycystic ovarian syndrome. She had no significant abdominal surgical history. Transvaginal ultrasound during evaluation revealed multiple FIGO type 2 fibroids and a small L ovarian endometrioma. The decision was made to proceed with video laparoscopic treatment of endometriosis with myomectomy and ovarian cystectomy. Upon surveying the abdomen, it was noted that in addition to fibroids and the ovarian endometrioma, there was evidence of severe adhesions of the large and small bowel with complete posterior cul-de-sac obliteration on the rectum and rectosigmoid colon. There were severe adhesions and fibrosis of the anterior and posterior cul-de-sac, rectal wall, rectovaginal septum, serosa of both ureters, rectosigmoid colon, and serosa of the internal iliac artery and vein as shown in Scheme 3. Endometriotic lesions were deeply infiltrative, involving approximately one-third of the lumen of the bowel and 6 cm length of the bowel. Due to the location of the affected area, shave excision was determined to be the most appropriate approach. Special care was made to provide minimal dissection of the retrorectal space and lateral pelvic sidewalls to decrease the risk of superior and inferior hypogastric plexus and sympathetic and parasympathetic nerve injuries. The ultimate goal was to restore anatomical structure while maintaining bowel integrity [2].

## 2. Introduction

Endometriosis is a systemic chronic inflammatory disorder that is predominantly modulated by estrogen. It affects roughly 10% of women in their reproductive years and can be observed in 80–90% or more of individuals who suffer predominantly from pelvic pain and infertility [3,4,5]. The condition can be classified into genital and extragenital forms, with endometriosis affecting the intestine being the most prevalent among the extragenital subtypes [6,7]. Colonic involvement can present as lesions located deeply within the muscular or mucosal layers of the colon, or as superficial disease that affects the serosal or subserosal portion of the bowel. The prevalence rate of intestinal endometriosis is between 3.8% and 37%, with the variation attributed to differences in classification and potential instances of underdiagnosis [8,9,10]. Bowel endometriosis warrants careful consideration in differential diagnoses due to its prevalence and the potential consequences of misdiagnosis. Delays in accurate identification can prolong the time to effective management, adversely affecting patients’ quality of life. Furthermore, delayed diagnosis may lead to disease progression, thereby increasing the risk of complications. In our experience, confirming the diagnosis can empower patients, offering them a greater sense of control over their condition. Given the systemic nature of endometriosis, a multidisciplinary approach is essential for optimal management. As there is no consensus and standard guideline for management, this review aims to provide a structured framework for the evaluation of bowel endometriosis, encompassing screening, diagnosis, and treatment. It also presents a comprehensive overview of available surgical techniques, guiding clinicians in the selection of the most appropriate approach tailored to each patient’s needs.

In conducting this narrative review, we searched multiple databases, including PubMed and Google Scholar, for studies addressing the management of bowel endometriosis. The review primarily focused on articles published since 2018, though older studies were included for context where relevant. Studies were included if they explicitly addressed management strategies, were peer-reviewed, and reported outcomes for patients with bowel endometriosis. Studies were excluded if they focused on non-bowel endometriosis, were not peer-reviewed, or used designs that lacked appropriate methodological rigor.

## 3. Etiology

The majority of patients diagnosed with intestinal endometriosis also present signs of the illness in other locations [7]. The primary etiology of bowel endometriosis could be similar to that of endometriosis in other sites. Numerous theoretical frameworks have been postulated to elucidate the complex mechanisms behind the pathophysiology of bowel endometriosis, a condition that is believed to involve multiple factors. These include retrograde menstruation and implantation of endometrial tissue outside of the uterine cavity, benign metastasis of endometrial tissue through uterine lymphatic drainage, coelomic metaplasia/embryonic rest, iatrogenic, immune dysregulation, endometrial stem cell recruitment, bone marrow-derived stem cell, hormone imbalance, epigenetic dysregulation, microRNA, carcinogenetic pathways, external environment, and lifestyle [7,11,12,13,14,15,16,17,18,19,20,21,22,23,24]. Nezhat and Mahmoud have proposed that the Allen–Masters peritoneal defect may serve as a conceivable pathway for the development of deep infiltrative endometriosis in cases of rectovaginal endometriosis [25]. Retrograde menstruation deposition can induce inflammation, thus increasing the likelihood of adhesion development and further cul-de-sac obliteration [26].

An emerging area of immune dysregulation in endometriosis is the relationship between bowel endometriosis and the oral/intestinal microbiome. Evidence suggests that dysbiosis of the intestinal microbiota disrupts immune homeostasis, driving chronic inflammation and creating a microenvironment conducive to the development and progression of endometriotic lesions [27]. Dysbiosis may impair anti-inflammatory pathways and compromise the intestinal epithelial barrier, thereby weakening mucosal integrity and facilitating bacterial migration, which worsens inflammation [28,29].

The intestinal microbiota also modulates estrogen metabolism via the estrobolome, influencing systemic estrogen levels and potentially contributing to the pathogenesis of endometriosis [30,31,32,33]. Alterations in the composition of the gut microbiome, including shifts in dominant bacterial taxa, may reflect systemic inflammatory states associated with the disease [27]. Furthermore, Toll-like receptor (TLR) pathways, which recognize bacterial components, have been implicated in the initiation of inflammatory responses that drive endometriosis progression [34,35]. 

These insights underscore the potential of the intestinal microbiome as a therapeutic target, with interventions aimed at restoring microbial balance and mitigating immune dysregulation offering promising avenues for the management of endometriosis.

## 4. Anatomy and Pathology

Endometriosis exhibits a predilection for the left colon. The rectosigmoid colon is the most commonly affected site, followed by the rectum, ileum, appendix, and cecum [36,37,38,39]. Isolated instances have also been documented in upper abdominal regions, such as the stomach and transverse colon [40,41]. Colonic involvement can present as lesions located deep within the muscular or mucosal layers of the colon, or as superficial disease that affects the serosal or subserosal portion of the bowel [8,10]. Bowel endometriosis has three distinct histologic subtypes: endometrial-like glandular mucosa, smooth-muscle-like tissue, and fibrotic tissue [42]. These histological variations reflect the complex nature of endometriosis’s pathology within the gastrointestinal tract. Moreover, within this intricate landscape, desmoplastic nodules emerge as a notable phenomenon, formed as a result of the host stromal cells responding to ectopic endometriotic implants by increasing the production of extracellular matrix proteins and collagen. The significance of this desmoplastic reaction remains uncertain [39].

The etiology of pain associated with endometriosis is complex and characterized by multiple factors. The existing data suggest an autonomic component playing a role in the manifestation of clinical symptoms, similar to those of irritable bowel syndrome [43,44]. Research findings indicate a potential association between endometriosis and certain chronic inflammatory bowel disorders [44]. Chiaffarino et al. conducted a meta-analysis which demonstrated that patients diagnosed with endometriosis exhibited a twofold-higher likelihood of developing irritable bowel disorder (IBS) compared to individuals without endometriosis [44]. When endometriotic lesions infiltrate the enteric nervous system, they can induce significant disruption. When they impact anatomical components such as the Auerbach plexus, Meissner plexus, or the interstitial cells of Cajal, individuals may manifest symptoms such as nausea, vomiting, or pain from subocclusive crises [45].

## 5. Cancer Risk

Although endometriosis is commonly perceived as a relatively harmless condition, it is linked to an increased susceptibility to cancer and increased obstetrical and non-obstetrical complications [46,47,48,49,50]. The estimated overall risk for the development of a neoplasm linked with endometriosis is up to 1% [46,49]. Among these cases, approximately 25% involve tissue that is located outside of the ovaries [49]. There have been documented instances of gastrointestinal tumors associated with endometriosis, with around 50% of these cases involving primary adenocarcinoma specifically in the rectosigmoid colon [50]. The precise mechanisms that contribute to the increased susceptibility to colorectal cancer in individuals with endometriosis remain incompletely understood. However, the available information indicates an increased likelihood of malignant transformation in individuals diagnosed with endometrioid or clear cell ovarian carcinoma [46,51,52]. As a result, excisional endometriosis surgery provides not only pain relief and the possibility to enhance fertility but is also a possible risk-reducing strategy against cancer [38].

## 6. Diagnosis

### 6.1. Medical History

The initial step in assessing for intestinal endometriosis requires conducting a thorough medical history. The patient’s history may reveal symptoms such as dysmenorrhea, profound dyspareunia, chronic pain, and dyschezia. Certain patients may also present with catamenial diarrhea, hematochezia, constipation, abdominal distension, discomfort with prolonged sitting, and extension of pain to the perineal region. The differential diagnosis involves a wide range of disorders, such as inflammatory or ischemic colitis, irritable bowel syndrome, pelvic adhesions, appendicitis, ovarian cysts, radiation colitis, diverticulitis, cancer, abscesses, or pelvic inflammatory disease [53,54]. The omission of bowel endometriosis in the diagnostic work-up can result in an extended period of misdiagnosis and a subsequent delay in treatment. Non-invasive screening methods may be an additional step to take for early diagnosis. These methods can include the Free Endometriosis Risk Advisor Application, BCL6 testing, endometrial function testing, and/or saliva testing [55,56,57,58].

### 6.2. Physical Examination

The physical examination of the vagina and rectum is particularly helpful when performed during menstruation, as this is when lesions may exhibit heightened inflammation, tenderness, and palpability. The pelvic examination may reveal a palpable nodule or an area of increased thickness along the uterosacral ligaments, uterus, vagina, or rectovaginal septum. A speculum exam may identify a laterally displaced cervix or the identification of a dark-blue lesion [59].

### 6.3. Serum Markers

The monitoring of CA-125 levels has been proposed as a diagnostic and disease progression assessment tool for profoundly infiltrative endometriosis; however, its practicality is limited, and it is not endorsed as a standard practice [60,61].

### 6.4. Other Non-Invasive Methods

Another possible non-invasive diagnostic tool is liquid biopsy, particularly through the analysis of microRNAs (miRNAs). miRNAs are small RNA molecules that regulate gene expression and are implicated in various cellular processes, including proliferation, apoptosis, and angiogenesis. In endometriosis, several miRNAs exhibit altered expression, making them valuable biomarkers for early detection. Ronsini et al. completed a systematic review that identified key miRNAs, such as miR-145, miR-451a, and miR-21-5p, with significant expression differences between endometriosis patients and healthy controls. miR-145 was highlighted for its upregulation in the early disease stages, suggesting its utility in identifying endometriotic lesions before the disease progresses [62]. Liquid biopsy enables miRNA extraction from minimally invasive samples like serum, plasma, or endometrial tissue, offering a cost-effective alternative to laparoscopy [62]. Furthermore, miRNAs may aid in monitoring therapeutic responses and predicting disease recurrence, contributing to personalized patient management. However, challenges remain, including variability in study methodologies and the need for miRNA-based assays’ validation [62]. Despite these hurdles, miRNAs hold great promise for revolutionizing endometriosis diagnosis, particularly in asymptomatic or mildly symptomatic patients, and may facilitate earlier interventions, reducing the diagnostic delay often associated with this condition.

In the context of the previously mentioned implications of the intestinal microbiome in endometriosis, [63]. discusses the possibility of the patient’s distinct stool metabolome being a mode of non-invasive diagnosis [63].

### 6.5. Diagnostic Imaging

The use of transvaginal ultrasonography (TVUS) in combination with a physical examination is a preferred approach, as it demonstrates a significant level of sensitivity and specificity. Imaging is essential for obtaining important lesion information related to size, location, depth of infiltration, presence of intestinal lumen stenosis, and quantification of nodules. These factors play a significant role in the preoperative planning phase. In 2011, Hudelist et al. conducted a comprehensive meta-analysis that demonstrated a notable specificity range of 92% to 100% for transvaginal ultrasound (TVUS), accompanied by a sensitivity range of 71% to 98% in the detection of bowel endometriosis [64]. In a similar vein, Exacoustos et al. documented a range of accuracy between 76% and 97% in their study, with the most notable accuracy (97%) reported in the identification of bladder lesions and cul-de-sac obliteration [65,66]. It is important to acknowledge that the precision of diagnosis is closely tied to the expertise of the sonographer. In order to address this, the International Deep Endometriosis Analysis group formulated recommendations to guide sonographers to achieve quality images [67]. One further constraint associated with transvaginal ultrasound (TVUS) is its potential inability to detect lesions located on the sigmoid, as they frequently lie beyond the scope of the imaging field [67]. While its diagnostic utility is limited, the application of enhanced transvaginal ultrasonography (TVUS) has been suggested as a potential aid in evaluating the extent of disease and identifying the presence of rectal stenosis [68].

When evaluating intestinal endometriosis, magnetic resonance imaging (MRI) is considered a secondary diagnostic tool, exhibiting higher specificity but lower sensitivity compared to transvaginal ultrasound (TVUS). MRI can serve as a valuable tool in corroborating suspicions of bowel endometriosis in cases when transvaginal ultrasound (TVUS) yields inconclusive results [69].

In 2015, a study conducted by Van der Wat and Kaplan examined the use of computed tomography-based modified virtual colonoscopy to detect the severity of bowel endometriosis. The test entailed introducing carbon dioxide into the rectum at a pressure of 25 mmHg, and subsequent computed tomography-guided pictures were taken to allow a three-dimensional representation of the gastrointestinal tract [68]. Despite being categorized as an experimental approach, this strategy exhibits encouraging initial findings. Other imaging modalities, such as a barium enema, may also be a helpful adjunct to the diagnosis of endometriosis lesions by assessing the extent of bowel occlusion [70]. Otherwise, intestinal endometriosis can also be detected as an incidental finding during an unrelated surgery.

## 7. Management

There are various interventions available for the management of intestinal endometriosis, encompassing both medical and surgical approaches. The selection of a modality is contingent upon the therapeutic objectives. Furthermore, the domains of medical and surgical management are not mutually exclusive and can be combined to achieve optimal symptom management.

### 7.1. Medical Management

The primary objective of medical management is to impede the progression of disease. It can be employed both before and after surgical intervention. It is important to recognize that medical intervention may be a temporizing measure for some and surgical treatment may still be required at a later stage. Ovulatory suppression may be advised for those who are not suitable candidates for surgical intervention or for those who have a preference to avoid it. Research has shown that hormonal suppression can offer substantial alleviation of pain and gastrointestinal (GI) symptoms in instances where bowel stenosis is below 60% [71]. After undergoing surgery, women who do not have an immediate desire to conceive may be given hormonal suppression treatment in order to prevent the recurrence of endometriotic lesions.

### 7.2. Current Hormonal Treatment Approaches

Currently, there is a lack of consensus about the most effective hormonal treatment regimen for the management or prevention of profoundly infiltrative endometriosis or intestinal endometriosis. The treatment principles prioritize the utilization of long-term hormonal suppression techniques while simultaneously limiting adverse effects in order to improve patient adherence. The initial medical therapy of choice is often low-dose progestins or combined oral contraceptives, mostly due to their high efficacy, few adverse effects, and cost-effectiveness [42].

### 7.3. Progesterone in Bowel Endometriosis

Progesterone plays a crucial role in the medical management of bowel endometriosis. Progesterone receptors have been found in both endometrial-like glandular lesions and smooth muscle lesions. Fibrotic tissue, however, does not exhibit their presence. The administration of progesterone has been shown to potentially impede the advancement of disease; nonetheless, it does not guarantee pain relief, particularly when symptoms arise from underlying structural changes [72].

Ferrero et al. conducted a study where they found that the administration of a modest dose of daily norethindrone (2.5 mg) resulted in a significant reduction in symptoms such as diarrhea, cramps, and cyclic rectal bleeding in women diagnosed with endometriosis [2]. Approximately 53% of the participants reported experiencing a notable improvement in gastrointestinal (GI) symptoms. Nonetheless, following the completion of the 12-month trial period, a significant proportion of patients, specifically 33%, elected to undergo surgical intervention for the management of their bowel endometriosis. This decision was primarily driven by the presence of persistent and distressing symptoms. The findings of a randomized controlled experiment conducted by Vercellini et al. demonstrated that the administration of progestins, either alone or in conjunction with low-dose estrogen, resulted in a considerable reduction in symptoms associated with dysmenorrhea, dyspareunia, and dyschezia [36,37].

### 7.4. Gonadotropin-Releasing Hormone Agonists

Leuprolide acetate, an agonist of gonadotropin-releasing hormone, has demonstrated efficacy in relieving symptoms associated with rectovaginal endometriosis in women. It may be administered in conjunction with add-back norethindrone therapy for a reduced side-effect profile. The utilization of leuprolide prior to surgery has the potential to diminish the overall disease burden during the surgical procedure. Nevertheless, the long-term utilization of gonadotropin-releasing hormone agonists is frequently limited due to their bothersome side-effect profile, which includes vasomotor symptoms.

### 7.5. Other Medical Interventions

Other medical interventions have been used with some success; however, supporting data have been sparse. Despite its limited sample size, a study by Fedele et al. showed that 11 women had significant improvements in dysmenorrhea, dyschezia, and pelvic pain following the use of a levonorgestrel intrauterine device [73]. In a study conducted by Razzi et al., it was observed that the administration of daily intravaginal danazol (at a dosage of 200 mg) was well tolerated by a cohort of 21 women diagnosed with rectovaginal endometriosis [74]. The treatment demonstrated a notable decrease in pain levels during the 12-month follow-up period without significant side effects. A summary of these medical therapy options is shown in Table 1.

### 7.6. Surgical Management

The primary objective of surgical therapy is the excision of endometriosis-affected tissue. The choice of the surgical approach to manage intestinal endometriosis is influenced by the surgeon’s level of expertise and access to suitable surgical tools. It is imperative to emphasize that surgical intervention should not be contemplated for patients who do not exhibit any symptoms. The management of bowel endometriosis often requires a multidisciplinary approach, comprising a gynecologic surgeon with expertise in minimally invasive procedures familiar with endometriosis and a gastrointestinal surgeon also familiar with endometriosis. This is because for decades, as early as 1986 and 1990, Dr. Camran Nezhat stated that “wherever in the body a cavity exists or can be created minimally invasive surgery is indicated and probably preferable. The limiting factor is the skill and experience of the surgeon and availability of proper instruments” [2,75,76,77,78,79].

### 7.7. Video Laparoscopic and Robotic-Assisted Surgical Approaches

Based on the surgeon’s level of expertise and availability of resources, we endorse the utilization of video-assisted laparoscopic surgery, either with or without the assistance of robotic arms. A multitude of research studies have consistently demonstrated a preference for video laparoscopy over laparotomy in the management of intestinal endometriosis [75,78,79,80,81]. Minimally invasive techniques have been found to be correlated with decreased blood loss, a shorter duration of hospitalization, and a lower incidence of postoperative problems. Laparoscopic operations, when performed with expertise, exhibit a relatively low rate of conversion to laparotomy, commonly estimated at approximately 3% [80]. A study conducted by Darai et al. employed a randomized controlled trial design to compare the long-term outcomes of laparoscopic-assisted and open colorectal resection procedures. The findings of this study revealed no significant differences in long-term outcomes between the two approaches. Additionally, the study suggested that video laparoscopy was associated with lower blood loss and a reduced incidence of adverse events. In a separate prospective trial conducted by Ruffo et al., it was found that laparoscopic resection led to a notably elevated postoperative pregnancy rate [77].

### 7.8. Classification of Surgical Technique

The surgical techniques employed for the treatment of bowel endometriosis can be classified into three main categories: shave excision, disc resection, and segmental resection. The selection of the appropriate approach is contingent upon various parameters, including the precise location of the lesion, the extent of infiltration, the quantity of nodules, and the presence of strictures [2,75,78,82,83,84,85].

The existing medical literature lacks definitive recommendations for determining the optimal surgical technique tailored to individual patient characteristics. There exist two predominant perspectives with regard to the selection of surgical technique. There are proponents who argue in favor of a more radical approach, which prioritizes total endometriosis lesion excision. As such, a considerable proportion of surgeons choose aggressive bowel resection as their primary treatment approach. While it has not been proven to achieve more favorable outcomes, it is important to acknowledge an increased risk of postoperative morbidity. The alternative viewpoint is to adopt conservative methods, such as shave excision and disc resection in order to reduce the occurrence of surgical morbidity. In light of the complex nature of this surgical challenge, the purpose of this expert review is to offer an overview to physicians who are faced with difficult decisions regarding the surgical management of intestinal endometriosis [82,83,84,85].

### 7.9. Shaving Excision

Shaving excision entails the gradual elimination of endometriotic lesions layer by layer, until the underlying healthy tissue is exposed, similar to peeling an onion. The objective of shaving excision is to remove the entirety or a substantial amount of the endometriotic and fibrotic lesions on the gut, while maintaining the structural integrity of the bowel mucosa and a segment of the muscularis if possible. Another goal is to reestablish the original anatomical structure of the soft tissue that may have been altered due to endometriosis. This technique is regarded as the most conservative method of endometriosis management [2,82,83,84,86].

### 7.10. Surgical Steps in Shaving Excision of Rectal Bulb and Rectosigmoid Colon

The main steps of the shaving procedure involve laterally identifying the ureter at a distance from the actual lesion. In cases when nodules measure more than 3 cm, 10% of them involve the ureter, necessitating ureterolysis, with or without the placement of a ureteral stent [82]. Upon the liberation of lateral gaps, the uterosacral ligaments are incised to maintain the connection between the bowel and the nodule. Shaving involves removing the nodule from the frontal region of the rectum in order to access the cleavage plane of the rectovaginal septum. However, there is a risk of unintentionally opening the intestinal lumen during this procedure. In this scenario, it is necessary to repair the bowel using staples or sutures [82,83]. To decrease the risk of entering the lumen of the bowel, we have always recommended starting with the right or left pararectal spaces and then reaching the rectovaginal space. Doing so, we first mobilize the rectum or rectosigmoid colon without any tension. The next step is shaving or disc-resecting the lesion [2].

The bowel lesion can be shaved using several methods such as the CO2 laser, cold scissors, ultrasound scalpel, plasma energy, or monopolar hook. Roman et al. completed a study that involved rectal shaving procedures in a sample of 54 women using plasma energy, and in another sample of 68 women using laparoscopic scissors. Two instances of postoperative rectal fistula development were documented. According to the findings reported by Roman et al., the outcomes of shaving excision were notably positive, as only a small proportion of patients (4%) encountered a return of symptoms. Furthermore, a pregnancy rate of 65.4% was recorded among patients who expressed the intention to conceive. Among these women, 59% achieved pregnancy through spontaneous conception [84].

The risk of significant complications is decreased following shaving in comparison to rectal resection. Rectovaginal fistulas, anastomotic leakage, delayed bleeding, and long-term bladder atony occur more frequently following rectal resection. Donnez and Roman conducted a review and found that certain complications were more common following rectal resection compared to the shaving approach. These complications include urinary retention (0–17.5%), ureteral lesions (0–2%), anastomotic leaks (0–4.8%), and pelvic abscesses (0–4.2%). The incidence of rectovaginal fistulas was greater following rectal resection (ranging from 0% to 18.1%) and disc excision (ranging from 0% to 11.6%) compared to shaving (ranging from 0% to 2.3%). If rectal resection is performed while lesions are positioned close to the anal verge and dentate line, the incidence of rectovaginal fistulas can increase up to 18%. Out of the 3298 cases who underwent shaving, only 0.06% of patients were found to have rectovaginal fistulas. Rectovaginal fistula rates are significantly lower when using shaving compared to other procedures, despite a small percentage (0–11%) of patients experiencing intraoperative bowel perforation with subsequent repair. The study conducted by Donnez and Roman found that the incidence of rectovaginal fistulas following shaving was just 0.25%, but the rates were approximately 2.8% and 4.3% after disc excision and rectal resection, respectively. Consequently, the risk seems to be mostly associated with removing a portion of the bowel rather than the vagina, particularly when dealing with lower lesions. Additionally, in 2005, Mohr et al. investigated a total of 178 female participants who had undergone laparoscopic intervention for profoundly infiltrative bowel endometriosis [79]. The interventions included shave excision (*n* = 93), disc excision (n = 38), and segmental resection (n = 47). The group that underwent segmental resection demonstrated a markedly elevated incidence of severe complications (*p* < 0.001). Within this cohort of patients, a proportion of 12.5% encountered diverse complications, encompassing ureterovaginal fistula, anastomotic stricture, intraoperative bladder perforation, rectal hemorrhage necessitating transfusion, and anastomotic leaking necessitating temporary colostomy. On the other hand, the group who underwent disc excision exhibited a lower incidence of serious sequelae, specifically 7.7%, which included pelvic abscess and rectovaginal fistula. There were no significant noteworthy issues documented among the individuals who underwent shave excision. Of note, the incidence of conception among infertile patients who underwent shaving or disc excision procedures was significantly greater compared to those who underwent segmental resection [81,84,87].

### 7.11. Efficacy and Long-Term Outcomes of Shave Excision

This procedure has received widespread endorsement from experts as a meticulous and exact approach for the appropriate management of extragenital endometriosis. Shaving excision is used for the removal of the deep endometriotic nodule in the context of deep infiltrative rectovaginal endometriosis [2,81,85,88]. The outcomes observed over an extended period of time subsequent to shave excision are particularly positive, and this specific strategy is characterized by the lowest occurrence of complications when compared to other surgical alternatives for the treatment of intestinal endometriosis. Dr. Camran Nezhat and his team have consistently documented exceptional postoperative outcomes since the 1980s [2,81,86,88]. Dr. Camran Nezhat and his team, in a cohort study of 185 women, ranging from 25 to 41 years old documented the outcomes of shave excision. In total, 162 out of 174 patients available for follow-up reported moderate to complete pain relief [2].

Initially reported in 1997 by Donnez et al., the most comprehensive and up-to-date investigation regarding surgical management of bowel endometriosis was conducted by the same researchers again in 2013. A total of 3298 procedures were performed for deep rectovaginal endometriotic nodules, mainly with the use of the shaving technique. The findings demonstrated a low level of complications; a single instance of rectal perforation, three instances of ureteral damage; and one instance of fecal peritonitis. In a prior study conducted by Donnez et al., encompassing a cohort of 500 patients who underwent rectovaginal shave excision, it was observed that 8% of the participants encountered recurring pelvic pain. Of the 388 patients included in this case series who expressed a wish to conceive, 57% achieved pregnancy through natural means, while 28% successfully conceived with the aid of in vitro fertilization [85,87].

### 7.12. Disc Excision

Laparoscopic disc excision, with or without the implementation of linear or circular staplers, for the management of bowel endometriosis has been extensively documented by our team and other researchers since the late 1980s [2,78]. This surgical method has gained widespread acceptance and is considered to be a practical option [89,90,91,92,93]. The procedure encompasses the entire excision of the affected portion of the intestinal wall, followed by the use of staples or sutures to close the ensuing opening. One crucial factor to take into account while contemplating disc excision is that the lesion should predominantly impact a restricted segment of the intestinal wall, often encompassing less than 50% of its greatest circumference [2,76,77,94].

The results subsequent to disc excision are highly favorable, exhibiting a notable reduction in postoperative complications in comparison to segmental resection, albeit with a slightly elevated risk of complications when compared to shave excision [76,77,80,89,95]. In 1994, we evaluated a cohort of eight female individuals, all of whom underwent disc excision as a treatment for intestinal endometriosis [76]. The mean duration of hospitalization was found to be three days, while the average size of the lesions was measured at 4.6 cm. Additionally, it is worth noting that one patient successfully obtained pregnancy [76]. Following that in 2016, Roman et al. completed a cohort study of 141 female individuals who received surgical intervention for endometriosis, namely involving the surgical removal of bowel lesions using laparoscopic disc excision. In this study, there were no instances necessitating the need for laparotomy, and the absence of postoperative sequelae, such as the creation of rectovaginal fistulas, ureteral injury, bowel perforation, or postoperative pelvic abscesses, is noteworthy. Within the initial month following surgery, a notable improvement in gastrointestinal and pain-related symptoms was observed in a substantial majority of the patient population, specifically 87% [80].

In 2016, an observational study was conducted involving a sample of patients who received three different surgical procedures: shaving (n = 47), disc (n = 15), and segmental resection (n = 30). A significant decrease in both short- and long-term pain, encompassing dysmenorrhea, dyschezia, and dyspareunia, was observed three months after the surgical procedure for all groups, as stated by the researchers. It is worth mentioning that patients who received shaving excision and disc resection exhibited a higher likelihood of experiencing symptom recurrence, which subsequently required reoperation, as compared to those who underwent segmental resection (27.6% for shaving, 13.3% for disc, and 6.6% for segmental). Despite the low sample size, the findings of this study indicate that disc excision may be a good middle ground; less morbidity with relatively favorable outcomes. However, it is important to note that the long-term durability of these effects may not be as significant as those achieved with segmental resection [96].

In 2011, Moawad et al. conducted a retrospective analysis to compare the outcomes of low anterior rectal disc resection (n = 8) with low anterior segmental resection (n = 14). The cohort of patients who underwent disc resection demonstrated some notable differences compared to the control group. Specifically, they experienced significantly shorter surgical durations (4 h against 7 h), lower levels of blood loss (134 mL versus 276 mL), and shorter durations of hospitalization (3 days versus 5 days). There were no difficulties occurring during the surgical procedure in either group. There was no significant difference observed in the size of the excised lesion between the two group. In contrast, it was seen that three patients in the cohort who underwent segmental resection experienced postoperative anastomotic strictures, out of which two patients necessitated further rectal dilation. Alternatively, the group that underwent disc resection did not experience any significant perioperative complications. Both groups indicated a significant degree of patient satisfaction following the surgical procedure. This study, albeit conducted on a limited sample size, indicates that both disc and segmental excision procedures can be efficacious in relieving symptoms. Nevertheless, disc excision is a surgical treatment that is characterized by its technical simplicity and reduced complication rate, especially when the lesion is situated in the lower regions of the digestive tract [97]. The outcomes can even be improved with the newest technology and increasing experience dealing with these complicated diseases.

### 7.13. Segmental Resection

Segmental resection of the affected bowel for the management of endometriosis was reported in the literature as early as 1907 [5,98,99]. Dr. Camran Nezhat and his team initially reported laparoscopic segmental bowel resection for bowel endometriosis in 1991 and 1992 as a therapy for bowel endometriosis [2]. Segmental excision entails the comprehensive segmental excision of a pathological portion of the gastrointestinal tract, followed by a surgical technique referred to as anastomosis. Segmental resection is commonly recommended for lesions that are extensive, circumferential, obstructive, or multifocal in nature. The re-anastomosis procedure can be conducted through either a primary end-to-end or side-to-side connection. Traditionally, segmental resection of endometriosis was completed via laparotomy. However, since the advent of video laparoscopy, a considerable number of skilled surgeons are now able to utilize minimally invasive procedures in order to obtain improved therapeutic results [26].

### 7.14. Outcomes and Complications of Segmental Resection

A systematic study of 1889 bowel resections for deep endometriosis was undertaken by De Cicco and colleagues in 2011. The mean duration of operation varied between 101 and 436 min, while the length of hospitalization ranged from 4 to 14 days. A significant proportion of women, namely 11%, experienced major difficulties during the course of the study. These complications encompassed several issues, such as a leakage rate of 2.7%, a fistula rate of 1.8%, a severe blockage rate of 2.7%, and a bleeding rate of 2.5%. While the evaluated studies exhibited inconsistencies in documenting the position of lesions, a significant number of problems were visibly linked to segmental resection in the lower rectum. More specifically, a higher probability of postoperative anastomosis leakage was seen with lower resections [100]. In a recent study conducted by Riiskjaer et al., a prospective analysis was conducted on a cohort of 128 patients who underwent segmental resection for bowel endometriosis. Although the findings show a significant long-term improvement in urine and sexual function one-year post-surgery, they also report a 7.4% incidence rate of anastomotic leakage [101].

### 7.15. Risks and Challenges of Radical Segmental Resection

Although the incidence of complications may be comparatively elevated in cases of segmental resection, it appears as though the likelihood of experiencing such complications is contingent upon the location of the lesion. Conversely, a growing contingent of surgeons emphasize the potential hazards linked to radical segmental resection and even the comparatively less invasive disc excision, especially when it entails substantial disturbance of the adjacent neurovascular structures. Extensive dissection of the pararectal and retrorectal space is necessary in cases of aggressive resection in the lower rectum region. This procedure poses a risk to important nerve bundles situated in this area, which may result in complications such as bowel stenosis, ischemia, severe constipation, bowel incontinence, and urine retention. Excisional procedures employed in other segments of the colon may present a lower incidence of serious complications and may be more commonly recommended. The prioritization of achieving total excision of endometriosis while considering the associated surgical risks is of the utmost significance [79,96,100].

### 7.16. Effectiveness of Segmental Resection Compared to Other Techniques

Segmental resection continues to be an essential approach for managing bowel endometriosis in certain situations, notably in individuals who continue to experience symptoms despite undergoing shaving or disc excision procedures. It is worth mentioning that De Cicco et al. documented that a significant proportion of patients who had segmental resection experienced total pain alleviation, with a success rate of 81.5%. Several studies indicate that shaving excision may not result in total pain relief, due to limited improvements in dysmenorrhea and dyspareunia. However, the research conducted by our group revealed that the rates of achieving total pain relief were significant not just in cases of segmental resection but also in relation to other surgical excision techniques. Specifically, the rates were found to be 80% after shaving excision, 95% following disc excision, and 89% following segmental resection [79,96,100].

### 7.17. Balancing Disease Eradication and Morbidity in Segmental Resection

The absence of clear guidelines may contribute to the continued occurrence of needless segmental bowel resections. We appreciate the aspiration to fully eradicate endometriotic lesions. Nevertheless, it is imperative to strike a delicate equilibrium between eradicating a benign disease and the subsequent morbidity in order to uphold the principle of “do no harm” as outlined in the Hippocratic oath. Bowel resection procedures increase patients’ morbidity, such as the possible requirement for a permanent ostomy, for a condition that may have been addressed with less invasive methods. However, in the instances where segmental resection is the appropriate action, it is advisable to use a multidisciplinary strategy that includes the expertise of a gastrointestinal surgeon or a gynecologic oncologist who possesses the necessary training in bowel resection. Segmental bowel resection should be avoided as much as possible the closer the lesion is to the rectal bulb and dentate line.

## 8. Considerations

### 8.1. Nerve-Sparing Surgery

**Figure 1:**
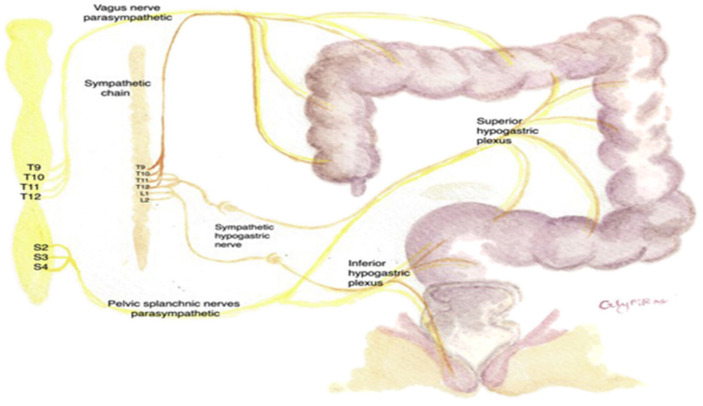
Bowel innervation.

**Figure 2:**
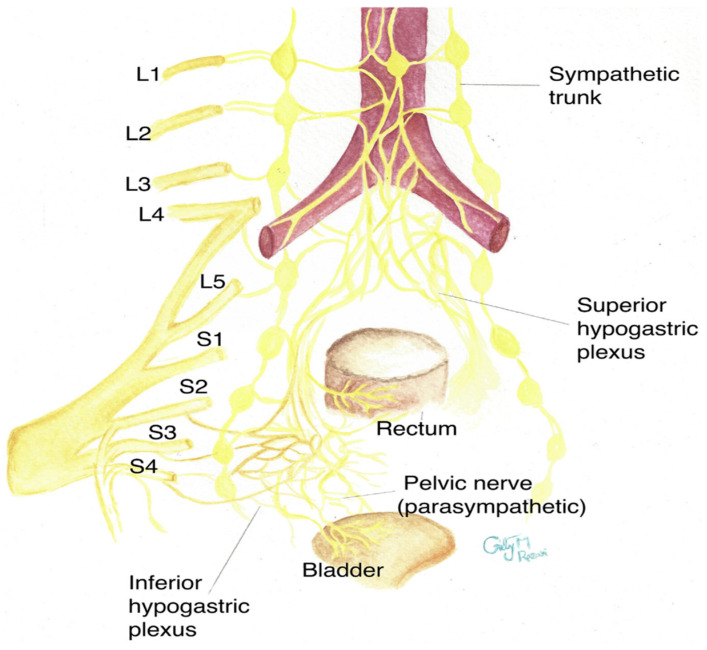
Bowel innervation. Reprinted/adapted with permission from Elsevier *American Journal of Obstetrics and Gynecology, Volume 218/Issue 6*, Camran Nezhat, MD, FACOG, FACS; Anjie Li, MD; Rebecca Falik, MD; Daniel Copeland, MD; Gity Razavi, MD; Alexandra Shakib, BS; Catalina Mihailide, BA; Holden Bamford, BA; Lucia DiFrancesco, MD; Salli Tazuke, MD; Pejman Ghanouni, MD, ACS; Homero Rivas, MD, FACS; Azadeh Nezhat, MD, FACOG; Ceana Nezhat, MD, FACOG, FACS; Farr Nezhat, MD, FACOG, FACS, *Bowel Endometriosis*, pp. 549–562. Copyright 2018.

The surgeon’s ability to avoid affected nerves may have an impact on the complication rate while performing shave, disc, or segmental resection of bowel endometriosis. Deeply infiltrative endometriosis has the capability to infiltrate both the superior and inferior hypogastric plexus, in addition to the sympathetic and parasympathetic nerve bundles (see Figure 1 and Figure 2). The impairment of these anatomical components has the potential to exacerbate symptoms related to the reproductive system, the genitourinary system, and the gastrointestinal system, hence exerting a detrimental impact on an individual’s overall well-being [102,103,104]. Postoperative urinary tract disease following such procedures is frequently anticipated, mostly due to the disruption of the nerve plexus, particularly the hypogastric plexus [105,106,107]. Consequently, many nerve-sparing strategies have been implemented in order to save the functionality of the intestine, bladder, and sexual organs [101,102,103,104]. The Tokyo method is a nerve-sparing technique that has demonstrated success [108]. During this procedure, the surgeon carefully divides and ties off the vascular section of the cardinal ligament, while ensuring the preservation of the branches of the pelvic splanchnic nerves [108]. Of note, Possover et al. employed electrostimulation as a method to detect and safeguard these nerves. Nevertheless, advanced pelvic disease is associated with a higher likelihood of dense nervous plexus engagement, which could hinder the preservation of the nerves [109].

The long-term outcomes of nerve-sparing procedures in the context of intestinal endometriosis treatment are currently limited in terms of available evidence; however, they generally show positive results. Ceccaroni et al. conducted a prospective study at a single center involving 126 patients to investigate the efficacy of the nerve-sparing technique in the surgical excision of bowel endometriosis [105]. The study findings revealed a decreased occurrence of bowel and bladder dysfunction, along with increased levels of patient satisfaction. Furthermore, the nerve-sparing technique demonstrated comparable rates of intraoperative complications when compared to conventional surgical methods for bowel endometriosis excision. Nerve-sparing approaches show promise, but further investigation is required to facilitate the broader adoption of this practice [105].

### 8.2. Determining Method of Surgical Intervention

The choice of a surgical method for symptomatic patients depends on various factors, such as the patient’s presenting symptoms and the anatomical location, size, and depth of the endometriotic bowel lesion [75,78,82,84,85]. To establish a structured framework for the approach, a classification system is utilized to categorize lesions based on their specific anatomical location. The physiological connections between the sigmoid colon and the peritoneal reflection along the left pelvic sidewall serve as important landmarks. Lesions are divided into four groups: (1) lesions located above the sigmoid colon, (2) lesions found on the sigmoid colon itself, (3) lesions affecting the rectosigmoid colon, and (4) lesions impacting the rectum. In addition to location, considerations include the size of the lesion, the degree of involvement (especially if the endometriotic lesion compresses or infiltrates the intestinal lumen), and the extent of invasion into the intestinal wall [83].

Whenever feasible, the selected procedure should aim to provide minimal dissection of the retrorectal space and lateral pelvic sidewall (Table 2). There is a potential risk of interrupting the superior and inferior hypogastric plexus, as well as the parasympathetic and sympathetic nerve branches, and the local blood supply while dissecting these regions. The aforementioned complexities may lead to persistent autonomic dysfunction of the gastrointestinal and urinary systems, potentially necessitating extended periods of self-catheterization or the permanent implementation of a colostomy. The risk increases as lesions move closer to the dentate line and anal verge. Lesions above the sigmoid colon are associated with the lowest complication rates. Specifically, dissection of the retrorectal, retrovaginal, pararectal, and paravesical spaces increases the likelihood of ureterovaginal fistula, anastomotic stricture, genitourinary difficulties during surgery, rectal bleeding that may require a blood transfusion, and anastomotic leakage that may necessitate a temporary ostomy. In instances of profound disease, it is possible that nerve entanglement may be inescapable, and the total removal of affected tissue may unavoidably lead to the impairment of these anatomical components. However, we firmly endorse the use of a prudent strategy, giving precedence to conservative surgical interventions wherever they are viable. The possible hazards linked to the radical excision of colorectal endometriosis should not be worse than the presenting symptoms of the patient [98,109,110,111,112].

### 8.3. Lesions Superior to the Sigmoid Colon

Segmental resection is the suggested surgical treatment for lesions located in the distal small bowel, ileocolic area, right hemicolon, and appendix. This approach is favored due to its relative ease and limited risk of nerve injury. It is important to highlight that in cases when endometriosis is found along the gut, an appendectomy may be performed even if no apparent disease is observed on the appendix. This is justified by the significant occurrence of occult appendicular endometriosis [113,114,115].

The preferred method for multifocal lesions or those that surpass 3 cm in size is segmental resection with a tension-free anastomosis. It is often recommended in cases when the lesions affect over one-third of the lumen of the upper gut. Alternatively, disc excision may be a viable option for lesions that are smaller than 3 cm, regardless of their involvement with the intestine lumen. Based on our empirical observations, it is evident that the utilization of a linear stapler for laparoscopic disc excision offers a comparative alternative, characterized by a diminished occurrence of leakage issues, mitigated perioperative pain, and lower morbidity [83].

Circling back to Case 1 where the ilieocecal area demonstrated an endometriotic lesion greater than 3 cm and constricted greater than one-third of the lumen, a tension-free anastomosis was recommended.

### 8.4. Lesions on the Sigmoid Colon

When encountering lesions located in the sigmoid region, it is crucial to restrict retrorectal dissection in order to mitigate the potential for enduring problems. Both segmental resection at or below the sigmoid region and disc excision, which involves lateral and posterior bowel mobilization, have been associated with a notable risk of surgical-site leakage after the operation. Additionally, these procedures have been linked to long-term bowel and bladder dysfunction, which could potentially result in the need for a permanent colostomy or permanent rectal incontinence and dyspareunia. Therefore, the preferred approach for managing sigmoid colon disease is shave excision. When considering dissection beyond the sigmoid colon, it is frequently unnecessary to extensively disrupt the retroperitoneum, thereby minimizing the potential for injury to the nerve and vascular plexuses. It is recommended that dissection be conducted mostly along the antimesenteric surface of the colon in order to preserve the vascular and nerve plexuses that are situated within the mesentery [98,105,106].

### 8.5. Lesions Affecting the Rectosigmoid Colon

Segmental resection of the rectosigmoid colon can be performed using the natural orifices of the rectum or vagina [80,116]. However, this method requires significant lateral mobilization and entry into the retrorectal region, which may result in postoperative problems. In order to mitigate these potential hazards, the preferred approach is to employ shave excision wherever feasible, especially for lesions over 3 cm in size, unless previous surgical interventions have proven ineffective. While the option of disc excision may be contemplated, it is advisable to exercise prudence in its approach. The Rouen procedure, which utilizes a trans-anal approach for the resection of extensive lesions in the disc, has been proposed as a viable alternative [115]. Complications that may arise subsequent to disc excision include pelvic abscess and rectovaginal fistula, albeit at a reduced incidence compared to segmental resection. The likelihood of these complications escalates as the dissection continues further downwards. Our inclination is to choose shave excision as the best method due to the increased postoperative risks associated with more aggressive treatments. Insufficient empirical data exist to support that the advantages of segmental resection surpass the associated risks in comparison to conservative surgery at this specific level, particularly in cases involving lower endometriotic lesions. The aforementioned lesions may not be as easily reachable using a linear stapler and performing extensive intestinal dissection can result in notable neurological and vascular consequences, as previously discussed [79,116,117].

### 8.6. Lesions Affecting the Rectum

In the context of rectal surgery, the objective of employing the shaving technique is to remove a substantial amount of pathological tissue while ensuring the integrity of the intestinal lumen and limiting lateral dissection in order to preserve the sympathetic and parasympathetic neural plexus. The decision to shave endometriosis off the rectum instead of risking intestinal perforation should be based on a comprehensive risk–benefit dialogue with the patient regarding their symptoms and goals, especially for individuals who are not actively wanting to conceive. In the case of women who do not express a desire to maintain their fertility, it may be prudent to explore the option of bilateral salpingo-oophorectomy, with or without hysterectomy, as an alternative to pursuing invasive rectal surgery. It is imperative to underscore that infertility should not be seen as a valid rationale for pursuing extensive intestinal surgery. Indeed, it has been seen that successful pregnancies can frequently be achieved even in instances of severe disease accompanied with intestinal stricture that has been managed through the utilization of the shaving procedure [2,80,85,96,118,119]. A considerable reduction in rectal endometriosis has often been reported in a certain group of individuals who receive second-look video laparoscopy after giving birth, exceeding the extent that can be attributed solely to shaving [83]. The cause for this regression is not yet fully understood, as there are other variables that can impact the results after surgery, making it difficult to conclusively assign these changes only to the surgical intervention. This finding may potentially reflect the perplexing characteristics of endometriosis. Among these factors are environmental, autoimmune, and nutritional factors [79,120,121,122].

### 8.7. Management of Incidental Finding of Bowel Endometriosis

In cases when bowel lesions are fortuitously detected during a separate surgical intervention, it is generally not recommended to undertake substantial dissection during the primary procedure, particularly if the patient presents with just mild gastrointestinal symptoms. Surgeons who possess expertise in the technique of shaving excision are capable of safely extracting lesions that can be effectively excised, thereafter submitting them for histological investigation. This methodology not only serves to validate the existence of bowel endometriosis in patients experiencing symptoms, but it may also offer comprehensive alleviation of the patient’s symptoms, while simultaneously excluding the possibility of malignancy. In cases when a patient’s symptoms continue to endure, it is justifiable to strategize for a prospective surgical intervention in conjunction with the involvement of a multidisciplinary group, which encompasses the expertise of a gastrointestinal surgeon and a urological surgeon, if necessary.

### 8.8. Complications

Complications are an intrinsic component of a surgeon’s professional reality, particularly when confronted with sophisticated surgical procedures. The more experienced the surgeon and his team are and the higher their surgical volume, the lower the risks of surgery. The historical data indicate a notably low incidence of unfavorable results, and we have additionally mitigated the likelihood of difficulties by refraining from employing invasive surgical procedures in the vicinity of the distal rectum. Nonetheless, it is imperative for surgeons to possess the necessary readiness to accurately detect and effectively handle a wide range of postoperative problems that may arise in the context of intestinal endometriosis surgery.

When seeking informed consent from patients prior to surgery, it is crucial to ensure that they are adequately informed about all of their alternative options, benefits, and risks. This should be a shared decision. In the case of segmental bowel resection, it is important to address potential perioperative problems, such as the occurrence of strictures, blockages, infections, perforations, fistulas, anastomotic leaks, and perioperative bleeding [96,98,108]. Although there is always a potential for intestinal perforation and leakage in any bowel operation, the danger is significantly reduced when using superficial shave excision. The utilization of appropriate surgical methods plays a crucial role in guaranteeing adequately vascularized and tension-free anastomoses, thereby reducing the probability of an anastomotic leak [7,78,100,116]. Additionally, the experience level of the surgeon should be a factor.

In order to optimize the process of postoperative recovery, the recommendation is the implementation of the enhanced recovery after surgery (ERAS) protocol (12). Additionally, we emphasize the importance of maintaining a regular connection with patients, utilizing daily telephone check-ins and in-office examinations as necessary. Postoperatively, each consecutive day should demonstrate a discernible improvement in the patient’s symptoms as a whole, and so we present a succinct compilation of potential complications that may arise after a surgical procedure, along with corresponding recommendations for their appropriate management throughout the postoperative period.

## 9. Conclusions

Deeply infiltrative endometriosis involving the bowel may exhibit diverse presentations. Unfortunately, the accurate identification of this condition frequently remains elusive. Alternatively, when diagnosed by less-experienced surgeons, it may be overtreated with aggressive therapeutic interventions. Bowel endometriosis can be discovered as an unintended finding during surgery conducted for unrelated purposes, or it may be suspected when a premenopausal female patient experiences significant pelvic pain, bloating, cyclic dyschezia, hematochezia, alterations in stool size, or symptoms resembling irritable bowel syndrome. In asymptomatic or mild cases, our strategy prioritizes diligent observation through the use of prolonged hormonal ovarian suppression, rather than surgical intervention.

In the case of symptomatic patients who have contraindications to medical therapy or who have experienced minimal improvement or recurrence from medical interventions, a surgical approach is preferred. The surgical strategy should incorporate the expertise of gynecologic and gastrointestinal specialists who possess extensive knowledge in the management of bowel endometriosis. There are surgeons who support the use of segmental resection of the intestine as the main approach for treating endometriosis, regardless of its specific location within the bowel. We disagree with this approach. The recommendation for shave excision is based on a thorough evaluation of the existing literature, combined with our significant experience in the field [82,84,86,88]. Shaving excision is generally preferred, especially for lesions located below the sigmoid colon, given the ability to avoid considerable lateral mobilization and dissection of the lateral and retrorectal regions. This approach helps to decrease potential negative impact on long-term bowel and bladder function. Patients exhibit exceptional outcomes and notable levels of satisfaction subsequent to undergoing shave excision [2,82,84,86,88]. Moreover, the incidence of complications related to shaving excision is comparatively minimal when compared to surgical alternatives, and it is accompanied by positive long-term results. As a result, the shaving procedure is utilized as the preferred approach for the management of endometriosis situated below the sigmoid colon, specifically targeting lesions in the lower rectum [2,82,83,84,86,88]. When dealing with lesions located proximal to the sigmoid colon, including those in the small bowel, segmental resection or disc resection are commonly chosen as the recommended surgical approaches.

The strengths of this review lie in its comprehensive evaluation of both surgical and medical management strategies for bowel endometriosis, with a focus on minimally invasive techniques. The multidisciplinary approach and inclusion of illustrative case studies further enhance its clinical relevance. However, we acknowledge that this review has limitations, such as the reliance on retrospective case series and the lack of long-term outcome data for some surgical approaches.

In summary, comprehensive management of bowel endometriosis requires a multidisciplinary, evidence-based approach that integrates advanced tools like transvaginal ultrasound and MRI, tailored surgical interventions, and postoperative strategies to minimize recurrence. Shaving excision is the preferred method for managing lower-bowel lesions, offering a balance between effective treatment and reduced morbidity. The refinement of nerve-sparing techniques and minimally invasive methods continues to enhance patient outcomes, aligning with the principle of “do no harm.” Future research should focus on optimizing surgical strategies and innovating less invasive approaches to further improve care for this complex condition.

## Figures and Tables

**Table 1:** Medical intervention.

Therapy	Indication	Key Studies/Findings	Limitations
Low-dose Progestins	Suppresses ovulation and reduces endometrial growth by acting on progesterone receptors	53% reduction in GI symptoms, though 33% opted for surgery after 12 months [72]	33% of patients may require surgery after long-term use due to persistent symptoms [72]
Combined Oral Contraceptives (OCP)	Prevents ovulation, reduces menstrual flow, and thins the endometrial lining	Reduces dysmenorrhea, dyspareunia, and dyschezia [36,37]	Potential side effects include weight gain, mood changes
Gonadotropin-Releasing Hormone (GnRH) Agonists	Suppresses ovarian hormone production by downregulating pituitary GnRH receptors	Leuprolide acetate with norethindrone add-back therapy mitigates side effects	Side effects such as vasomotor symptoms limit long-term use
Levonorgestrel Intrauterine Device (IUD)	Releases levonorgestrel locally to inhibit endometrial growth and reduce symptoms	Significant reduction in dysmenorrhea, dyschezia, and pelvic pain [73]	Limited supporting data, small sample sizes
Intravaginal Danazol	Androgenic effects suppress estrogen production and decrease endometrial tissue proliferation	Intravaginal administration of danazol reduced pain with minimal side effects [74]	Limited sample size, requires further research

**Table 2:** Management of endometriotic lesions based on location.

Lesion Location	Preferred Procedure	Indication	Alternative Options	Complications/ Special Considerations
Lesions Superior to the Sigmoid Colon	Segmental resection	Lesions > 3 cm or involving > 1/3 of the lumen in the distal small bowel, ileocolic area, right hemicolon, or appendix	Disc excision for lesions < 3 cm	Appendectomy recommended even without visible disease (occult endometriosis)
Lesions on the Sigmoid Colon	Shave excision	Sigmoid lesions where extensive retroperitoneal dissection is unnecessary		Avoid extensive retrorectal dissection to minimize nerve/vascular injury; long-term bowel/bladder dysfunction with more aggressive approaches
Lesions Affecting the Rectosigmoid Colon	Shave excision	Lesions > 3 cm, unless prior surgery was ineffective	Disc excision (use with caution)	High risk of complications (rectovaginal fistula, pelvic abscess) with more aggressive techniques like segmental resection
Lesions Affecting the Rectum	Shave excision	Conservative approach for women not seeking immediate fertility	Bilateral salpingo-oophorectomy, hysterectomy for non-fertility-seeking patients	Radical rectal surgery should be avoided unless necessary (in the case of lesion involving over one-third of the bowel lumen), as infertility is not a strong justification; successful pregnancies are still possible with conservative surgery
